# Bilateral symmetrical lymphangiomas of the gingiva: case report

**DOI:** 10.1186/1746-1596-1-9

**Published:** 2006-05-20

**Authors:** Pouria Motahhary, Babak Sarrafpour, Afshin Abdirad

**Affiliations:** 1 Department of Oral Pathology, Dental School, Tehran University of Medical Sciences, Iran; 2 Department of Pathology, Cancer Institute, Tehran University of Medical Sciences, Iran

## Abstract

**Background:**

Lymphangioma is a benign lesion that is related to proliferation of lymphatic vessels. Lymphangioma of the gingiva is a rare lesion that manifest as a pebbly hyperplasia on gingiva. The purpose of this study is to report a case of bilateral symmetrical lymphangioma of the gingiva.

**Case presentation:**

A 32-year-old man was presented with bilateral hyperplasia of gingiva in upper canine regions. The lesions were resected completely and evaluated histologically. The microscopic evaluation revealed lymphangioma.

**Conclusion:**

Bilateral lymphangioma of the gingiva is a very rare lesion which its origin is controversial.

## Background

The lymphangioma is a well-known benign hamartomatous tumor of lymphatic vessels, which have a marked predilection for head and neck [1]. Oral lesions are most frequently found on the tongue and usually demonstrate a pebbly appearance as by their superficial location. Occurrence in other areas such as cheeks, lips, floor of the mouth, palate and gingiva has been reported [2]. Congenital alveolar lymphangioma is seen as a unique lesion on the alveolar mucosa of African-American neonates [1].

Two unusual cases of bilateral symmetrical lymphangiomas of the gingiva have been reported by Josephson and van Wyk [3] and McDaniel and Adcock [4]. The following report is another case of bilateral symmetrical lymphangioma of the gingiva which occurred in anterior maxillary gingiva as areas of hyperplastic gingivitis.

## Case presentation

A healthy 32-year-old man was referred to the Department of Periodontology of Tehran University of Medical Sciences complaining of gingival hyperplasia on the lateral and canine teeth of maxilla in both sides. The past medical history was unremarkable. In clinical examination clear vesicular asymptomatic lesions were found on the attached and marginal gingiva in canine area. He had no previous dental history relating to the lesion in those sites and lesions were appeared gradually from two years ago. No other lesion and alteration could be detected radiographically and clinically. Excisional biopsies were taken from both lesions and the specimens were sent to the Department of Oral Pathology of Tehran University of Medical Sciences for microscopic examination.

**Figure 1:**
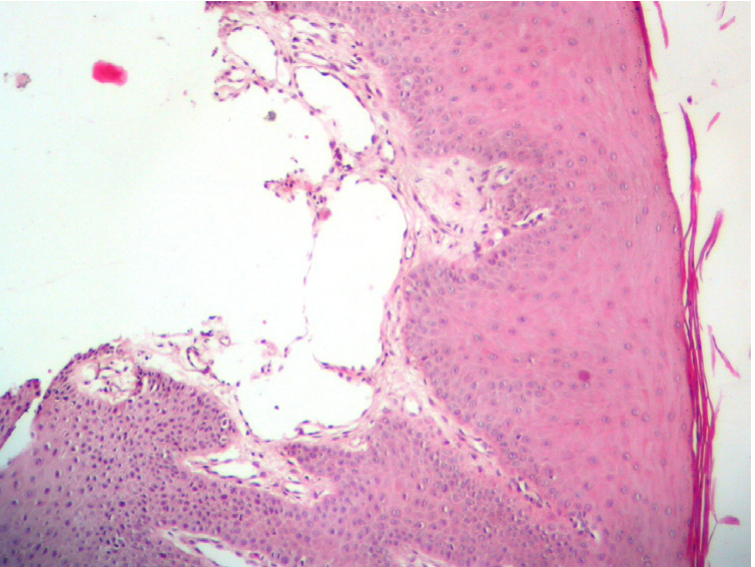
Microscopic appearance of the lymphangioma of right side, the dilated lymphatic channels lined by endothelial cells can be observed.

**Figure 2:**
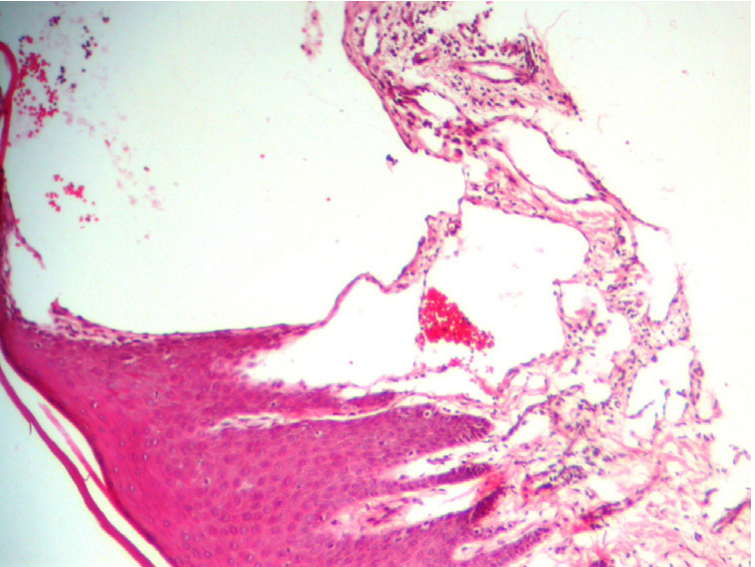
Microscopic appearance of the lymphangioma of left side, the dilated lymphatic channels lined by endothelial cells can be observed.

Microscopic evaluation revealed gingival tissue covered by parakeratinized squamous epithelium. Deep to the epithelium multiple dilated lymph vessels with different sizes were observed in a little loose fibrovascular tissue. These spaces were lined by a single layer of endothelial cells with flattened nuclei, and contained lymph with a few lymphocytes. Vessels just beneath the surface epithelium filled the connective tissue papilla. Both sides had similar appearance in histologic examination (figure 1 and 2). The diagnosis was lymphangioma.

The surgical wounds healed without scar formation in 4 weeks and no sign of recurrence was evident after 10 months.

## Conclusion

Microscopically lymphangioma consist of capillary or cavernous lymphatic channels, which lined by endothelium and appeared empty or filled with proteinaceous material and occasional lymphocytes. Specific antibodies against vascular endothelial cells are used for detection of microvesseles. Pan-endothelial markers such as CD34 (an antibody targeting the transmembranous sialo protein), CD31 (an antibody targeting the platelet-derived cell adhesion factor that is present in endothelial cells) and CD105 (endoglin) are generally used for identification of microvessels [5,6]. But none of these factors are specific for lymphatic vessels. Recently several relatively specific antibodies for lymphatic endothelium, such as VEGR3 (vascular endothelial growth factor receptor 3), podoplanin, lymphatic vessel endothelial HA receptor-1 (LYVE-1), Prox1 and D2-40 have been identified [7] and It has been demonstrated that podoplanin and D2-40 monoclonal antibody can be used as reliable lymphatic endothelial cell marker for distinguishing blood vessels from lymphatic vessels [8].

It is often difficult to state whether lymphangioma are true neoplasms, hamartomas or lymphangiectasias. In the first two forms it is a malformation that arises from sequestration of lymphatic tissue that fails to communicate normally with the lymphatic system and have some capacity to proliferation. Lesions designated as acquired lymphangiectasia may develop as a result of infection or surgery that interferes with regional lymphatic drainage [9].

Bilateral and symmetrical distribution of lesions in this case, in addition to lacks of infection or surgery (that may cause localized lymphatic obstruction) rule out the possibility of acquired lymphangiectasia. On the other hand confined growth potential and bilateral distribution suggests a hamartomatous nature and probably a developmental origin. This point of view is in agreement with hypothesis of previous researchers [3,4] but the time of onset (30 years old) seems to be a little high for developmental lesions.

Traditionally lymphangiomas were divided into three groups: simplex (capillary), cavernous and cystic [9]. Although Bill and Sumner [10] suggested that histological differences in various lymphangiomas are attributed to the differences in anatomic location and therefore that histologic classification is of little benefit [9,10]. In according to this concept some authorities classify the cutaneous lymphangioma into superficial and deep types [11]. From this point of view this lesion can be considered as superficial lymphangioma.

Finally it can be concluded that in rare instances bilateral symmetrical lymphangioma, confined to the superficial lamina properia of gingiva may become clinically apparent. It is not possible to definitely determine the origin of this lesion but due to its behavior and location it may have developmental origin.

## Competing interests

The author(s) declare that they have no competing interests.

## Authors' contributions

Pouria Motahhary and Babak Sarrafpour were participated in preparing the material and drafting the manuscript equally and Afshin Abdirad was involved in sequence alignment.
